# Michael Haschke, Jörg Flock, and Michael Haller: X-ray fluorescence spectroscopy for laboratory applications

**DOI:** 10.1007/s00216-021-03592-1

**Published:** 2021-08-25

**Authors:** Kerstin Leopold

**Affiliations:** Institute of Analytical and Bioanalytical Chemistry, Ulm University, 89081 Ulm, Germany

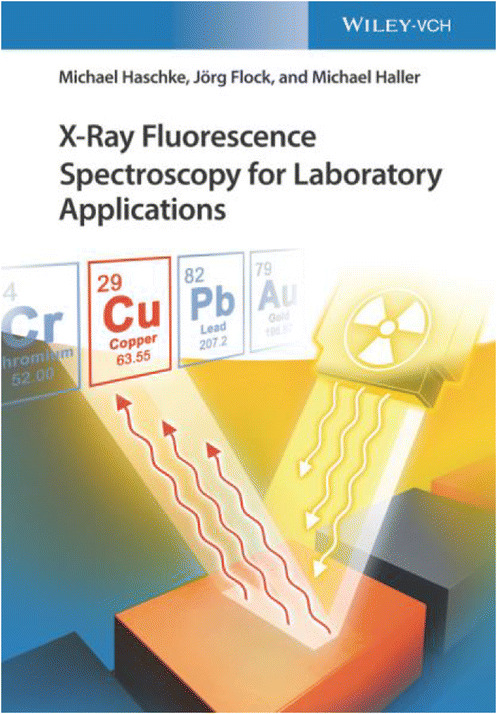



**Bibliography**

X-ray fluorescence spectroscopy for laboratory applications

Michael Haschke, Jörg Flock, and Michael Haller

ISBN: 978-3-527-34463-5

Wiley-VCH

Hardcover, 496 pages

2021, €139.00

## Book’s topic

X-ray fluorescence spectroscopy (XRF) is a versatile analytical technique providing qualitative and often also quantitative chemical element information. Depending on the instrumental set up, bulk analysis of solids and liquids as well as coating and layer analysis are possible. Trace element analysis is achieved in total reflection measurement condition, while element distributions can be investigated using micro-XRF. Several manufacturers offer diverse laboratory instrumentation ranging from bulky floor-standing equipment, to desktop instruments and down to hand-held devices. Consequently, a broad variety of different laboratory applications of XRF analysis have been establish in various fields within the last decades. The book *X-ray fluorescence spectroscopy for laboratory applications* provides a comprehensive overview of these applications and introduces to the method’s principles. It covers the different instrument types as well as it summarises sample preparation and data evaluation methods.

## Contents

The book comprises 19 chapters and an informative appendix. In the introduction, a brief outline on historical and recent development of XRF instruments and application fields is given. The second chapter presents the principles of X-ray spectrometry explaining also the fundamentals of X-ray radiation and their interaction with matter. Chapters 3 to 6 basically follow the analytical process and describe sample preparation, general design of X-ray spectrometers, measurement and evaluation of spectra, and finally analytical quality assessment. Other element analytical methods are summarised in chapter 7 and most important facts on radiation protection are presented in Chapter 8. Chapters 9 to 17 cover distinct analytical applications in details, like for instance analysis of powders, trace analysis or analysis of element distributions. The last two chapters present general information on process control and quality management, respectively. Finally, tabular display of relevant data and further important information is presented in the appendix.

## Comparison with the existing literature

The book can be regarded as English-language version of *Röntgenfluoreszenzanalyse in der Laborpraxis* published in 2017, written by Michael Haschke and Jörg Flock. In this new book, Michael Haller complements the experienced team of authors. Other books published within the last decade in this field rather focus on a particular instrument type, like *Laboratory micro X-ray fluorescence* (2014, Author: Michael Haschke) or application field, e.g. *X-ray fluorescence spectrometry (XRF) in geoarchaeology* (2011, Ed.: Shackley, M. Steven), than discuss comprehensively all aspects. Only the book entitled *X-ray fluorescence spectrometry and related techniques: An introduction*, part of a book series on Spectroscopy, published in 2013 and written by Eva Marguí and René van Grieken, also gives an overview on X-ray fluorescence techniques and applications. In comparison, *X-ray fluorescence spectroscopy for laboratory applications* gives more emphasis on laboratory applications and presents a greater number and broader variety of examples.

## Critical assessment

All three authors have been working with X-rays as an analytical tool for many years, have developed and introduced new techniques, instruments, devices and software. Their broad practical experience and theoretical insights are reflected in the comprehensive presentation of the subject. The focus of this book is clearly on application and examples. Consequently, the large number of examples and broad variety of analytical questions and applications is its greatest benefit. In addition, principles and theory of X-ray fluorescence are clearly presented and adequately discussed to provide fundamental understanding of the method. Figures, equations and tables are clearly arranged and help to understand the content.

## Readership recommendation

The book is addressed to analytical chemists, analytical laboratories, material scientists, environmental chemists, chemical engineers, biotechnologists, and pharma engineers. For practitioners in the field of X-ray spectrometry it is of high value and can also be used as a reference book. Furthermore, it can be recommended to researchers working with any type of X-ray fluorescence spectrometer and due to its clear presentation of principles and theory it may serve as a course book for bachelor and master students.

## Summary

*X-ray fluorescence spectroscopy for laboratory applications* is a strongly recommended, high-quality monograph in the field of X-ray spectroscopy. As the title announces, it emphasises on laboratory applications, but still, it presents principles and theory adequately and in sufficient detail. Therefore, it is a unique resource for practitioners and scientists.

